# Protection of Rhesus Monkeys by a DNA Prime/Poxvirus Boost Malaria Vaccine Depends on Optimal DNA Priming and Inclusion of Blood Stage Antigens

**DOI:** 10.1371/journal.pone.0001063

**Published:** 2007-10-24

**Authors:** Walter R. Weiss, Anita Kumar, George Jiang, Jackie Williams, Anthony Bostick, Solomon Conteh, David Fryauff, Joao Aguiar, Manmohan Singh, Derek T. O'Hagan, Jeffery B. Ulmer, Thomas L. Richie

**Affiliations:** 1 Naval Medical Research Center, Silver Spring, Maryland, United States of America; 2 Walter Reed Army Institute of Research, Silver Spring, Maryland, United States of America; 3 Chiron Corporation, Emeryville, California, United States of America; New York University School of Medicine, United States of America

## Abstract

**Background:**

We have previously described a four antigen malaria vaccine consisting of DNA plasmids boosted by recombinant poxviruses which protects a high percentage of rhesus monkeys against *Plasmodium knowlesi* (Pk) malaria. This is a multi-stage vaccine that includes two pre-erythrocytic antigens, PkCSP and PkSSP2(TRAP), and two erythrocytic antigens, PkAMA-1 and PkMSP-1(42kD). The present study reports three further experiments where we investigate the effects of DNA dose, timing, and formulation. We also compare vaccines utilizing only the pre-erythrocytic antigens with the four antigen vaccine.

**Methodology:**

In three experiments, rhesus monkeys were immunized with malaria vaccines using DNA plasmid injections followed by boosting with poxvirus vaccine. A variety of parameters were tested, including formulation of DNA on poly-lactic co-glycolide (PLG) particles, varying the number of DNA injections and the amount of DNA, varying the interval between the last DNA injection to the poxvirus boost from 7 to 21 weeks, and using vaccines with from one to four malaria antigens. Monkeys were challenged with Pk sporozoites given iv 2 to 4 weeks after the poxvirus injection, and parasitemia was measured by daily Giemsa stained blood films. Immune responses in venous blood samples taken after each vaccine injection were measured by ELIspot production of interferon-γ, and by ELISA.

**Conclusions:**

1) the number of DNA injections, the formulation of the DNA plasmids, and the interval between the last DNA injection and the poxvirus injection are critical to vaccine efficacy. However, the total dose used for DNA priming is not as important; 2) the blood stage antigens PkAMA-1 and PkMSP-1 were able to protect against high parasitemias as part of a genetic vaccine where antigen folding is not well defined; 3) immunization with PkSSP2 DNA inhibited immune responses to PkCSP DNA even when vaccinations were given into separate legs; and 4) in a counter-intuitive result, higher interferon-γ ELIspot responses to the PkCSP antigen correlated with earlier appearance of parasites in the blood, despite the fact that PkCSP vaccines had a protective effect.

## Introduction

In recent years, research on vaccines against malaria infection has been moving along two parallel tracks. On the one track are the recombinant protein vaccines given with novel adjutants. Human phase 1 and 2a/b trials of several of these vaccines have shown moderate levels of efficacy [1]–[10]. Great emphasis is placed on the proper folding and glycosylation of recombinant protein vaccines, particularly of the blood stage antigens, as protective antibodies often recognize conformational epitopes on these antigens. [11]–[23].

The other track of malaria vaccine development is genetic vaccination with DNA plasmids, recombinant viral vectors, or often with both in a DNA prime-viral boost combination. In rodent models, genetic vaccines produce strong T cell responses of both CD4^+^ and CD8^+^ subsets, as well as specific antibodies. The murine prime-boost vaccines have been very successful at attacking the malaria parasite as it develops inside the hepatocyte prior to infecting red blood cells [24]–[31]. Genetic pre-erythrocytic vaccines have also begun to be tested in human phase 1 and 2a malaria trials [32]–[44]. However, these initial studies show that genetic vaccines are less immunogenic in humans, with only slight efficacy in preventing parasitemia following experimental sporozoite infection.

Several years ago, we decided to develop a primate malaria vaccine model for sporozoite infection. This would allow us to study immunity against sporozoites, liver stage, and blood stage malaria in a model system closer to the human than the mouse, and to optimize the delivery of genetic malaria vaccines as a guide to planning human trials. We chose to use *Plasmodium knowlesi* (Pk) in the Indian rhesus monkey, as sporozoites are highly infectious and blood stage parasitemias rise to high levels similar to *P. falciparum* in humans. Our previous studies have shown that DNA prime-viral boost malaria vaccines can partially protect monkeys [45], [46]. Our vaccine regimen uses a mix of four Pk antigens (PkCSP, PkSSP2 (TRAP), PkAMA1, and PkMSP1-42kD subunit). The vaccine has typically used three injections of DNA in PBS for priming, followed by a recombinant COPAK poxvirus boost after a 16 week interval. This vaccine has protected approximately 60% of Indian origin rhesus monkeys against potentially lethal Pk malaria (10% sterile protection plus an additional 50% which have parasites in their blood but self-cure). In this paper, we will refer to this vaccine as DNA Pk4×3/COPAK, to emphasize that it uses four Pk antigens and has three doses of DNA before the recombinant COPAK poxvirus boost.

The three experiments presented in this paper all include the DNA Pk4×3/COPAK vaccine, and compare variations on the priming regimen or the number of antigens included. We hoped that some of the variations, such as the use of high doses of DNA plasmid or the use of DNA on poly-lactic co-glycolide (PLG) particles [47]–[50], might lead to better protection. Other variations, such as shortening vaccination intervals or priming with single DNA doses, were aimed at streamlining vaccine schedules. By comparing the four antigen vaccine with vaccines containing only the pre-erythrocytic components (PkCSP and PkSSP2) we hoped to understand the role of the different antigens in protection. Endpoints for all three experiments were parasitemia after sporozoite challenge and in vitro T cell and antibody responses. We begin by presenting each experiment separately, including parasitemias after challenge and immune responses. At the end of the paper, we combine results from all three studies to look at the correlation of immune responses with protection.

## Methods

### Animals

Rhesus monkeys (*Macaca mulatta)* descended from Indian stock were used for all three experiments. Animals were from 6 to 16 years old and of both sexes. For Experiment #1, monkeys were obtained by and housed at Southern Research Institute, Frederick MD. For Experiments #2 and #3, monkeys were obtained by and housed at the Walter Reed Army Institute of Research/Naval Medical Research Center Silver Spring, MD. Experiment #1 was approved by the SRI Institutional Animal Care and Use Committee. Experiments #2 and #3 were approved by the WRAIR/NMRC Institutional Animal Care and Use Committee. All experiments were conducted according to *Guide for the Care and Use of Laboratory Animals* 1996.

Animals were selected to be in general good health, and to have no history of prior exposure to malaria or pox viruses. Prior to selection for the studies serum specimens from all animals were tested in IFAT assays against Pk sporozoites and Pk infected red cells, and all animals with positive serum titers at dilutions of 1∶80 or higher were excluded.

### Plasmid vaccines

The DNA plasmid vaccines encoding Pk genes have been previously described [45]. Briefly, DNA sequences encoding the full length genes from the Pk H strain of PkCSP, PkSSP2, and PkAMA-1 and the 42 kD C terminal fragment of PkMSP-1 were cloned into the VR1020 mammalian expression vector (Vical Inc, San Diego CA). This vector contains a CMV promoter, and a TPA signal sequence. Each gene was cloned into a separate plasmid. DNA plasmids for vaccination were produced by Vical, Inc and contained less that 0.6 EU of endotoxin per mg and were at least 80% super-coiled. Plasmids were diluted in PBS pH 7.2 prior to injection.

PLG formulation of DNA plasmids. In Experiment #1 some animals were immunized with DNA plasmids adsorbed to poly-lactic co-glycolide (PLG) particles. Formulation of these DNA-PLG particles was as previously described [50]. Each of the 4 DNA plasmids was adsorbed to separate PLG particles. A dose equivalent to 0.5 mg of each plasmid was injected in a total volume of 1ml.

### Poxvirus

The poxvirus vaccines encoding Pk genes have been previously described [45]. Briefly, the same four Pk DNA sequences which were used to construct the Pk DNA plasmids were cloned into the COPAK poxvirus immunization vector (Virogenetics, Troy, N.Y). COPAK is derived from the Copenhagen strain of vaccinia virus. Each Pk gene was cloned into a separate COPAK virus.

### Immunizations

DNA injections

Experiments #1 and #3. DNA was diluted in PBS pH 7.2 and immunizations were given with a #20 gauge needle and syringe. PLG formulated DNA was injected using a #18 gauge needle because of increased viscosity. Each injection had a total volume of 1 ml and a total of 0.5 mg of plasmid. Each plasmid was injected separately and subsequent injections of each plasmid were into the same muscle: PkCSP right rectus femoris, PkSSP2 left rectus femoris, PkAMA1 right triceps, and PkMSP1 left triceps.

Experiment #2. Immunizations in this experiment were the same as in Experiments #1 except for the group receiving 5 mg of PkCSP DNA per injection. Because of increased viscosity, for this group the DNA was diluted in 2 ml of PBS, and 1 ml was injected into each femoral muscle.

COPAK injections were given im with a #20 gauge needle and syringe into the right rectus femoris muscle in 1 ml of PBS pH 7.2. All COPAK vaccinations used 2×10^8^ pfu of virus for each malaria antigen. For multiple antigen COPAK immunizations, all vaccines were mixed together in the same syringe in 1 ml of PBS and delivered into the same site, a total of 8×10^8^ pfu . Control COPAK for the Pk4 vaccine was 8×10^8^ pfu of parental COPAK virus.

### Malaria Parasites

*Plasmodium knowlesi* H strain sporozoites were grown in *Anopheles dirus* mosquitoes. Sporozoites were harvested 14 days after mosquitoes had fed on an rhesus monkey infected with Pk. Harvesting was by the Ozaki method. Sporozoites were diluted in E199 medium with 5% normal rhesus serum and counted with a hemocytometer. 100 sporozoites in a total volume of 1 ml were injected IV on the day of challenge.

Beginning 6 days after sporozoite challenge, each day at 1 PM blood was taken by ear prick and prepared for thin and thick malaria smears using Giemsa stain at pH 7.01. For thin smears, 20,000 red cells were examined. For thick smears, 0.025 µl of blood were examined. These data was used to calculate the percent infected red blood cells. Animals were followed for 30 days after challenge. To minimize morbidity, monkeys were treated when their parasitemia exceeded 1% in experiment #1. Because of the lack of morbidity in experiment #1 the treatment threshold was raised to 2% in experiments #2 and #3. Monkeys were also treated if their hematocrit fell to 50% of baseline values, which occurred in several animals with malaria infections which persisted at low levels for longer than 18 days after sporozoite challenge.

### Measurement of malaria antibodies

Plasma was tested by ELISA for IgG titer using as capture antigens each of the four Pk antigens used in the immunization studies. Capture antigen for PkCSP was an *E. coli* produced full length protein used at 0.1 microgram per ml provided by Sanjay Kumar. Capture antigen for the full length PkSSP2, PkAMA-1, and the PkMSP-1 42kD fragment we produced by in vitro synthesis using the Rapid Translation System RTS 500 E. coli HY kit (Roche Diagnostics Corporation, Indianapolis, IN). These capture antigens were used at concentrations of 1 to 4 micrograms per ml in PBS pH 7.2 in Immulon II 96 well plates (Dynex Technologies Inc., Chantilly, Virginia ). Plates were blocked with 5% milk powder in PBS for 2 hours. Plasma samples were diluted in 3% non-fat dry milk in PBS and kept at room temperature for 4–18 hours. Peroxidase-labeled goat anti-human IgG (H+L) (Kierkegard Perry Laboratories, Gaithersburg MD) at a 1∶10,000 dilution in 3% non-fat dry milk was added for 1 hour, and substrate was ABTS (Kierkegard Perry Laboratories). OD was read using a SPECTRA MAX 190 ELISA reader (Molecular Devices Corp., Sunnyvale, CA). Endpoint titer for each sample was the highest plasma dilution at which the OD was greater than twice the value of plasma from naïve monkeys.

### Measurement of T cell responses

ELIspot assay for cells producing INF-γ was done as previously described [51]. Briefly, MAIP S 4510 plates (Millipore, Bedford, MA) were coated with BMS 107, a monoclonal antibody against human interferon γ, (Bender Med Systems, Austria) at 5 µg/ml in PBS. 2×10^5^ cryopreserved PBMC were added per well in a total volume of 0.2 ml containing medium alone, concanavalin A at 10 microgram per ml or malaria antigen. After overnight incubation, a secondary anti-human biotinylated anti-IFN-γ antibody, clone 7B6-1 (Mabtech, Cincinnati, OH) was added at concentration of 2.5 µg/ml. Spots were developed using streptavidin -alkaline phosphatase conjugate (PharMingen, San Diego, CA) at 1∶2000 dilution at room temperature for 1 hr. Following six washes with PBS Tween, spots were developed with 5-bromo-4 chloro-3 indolyl phosphatase (BCIP) (Sigma-Aldrich, St. Louis, MO). Spots were read using a CTL ELIspot reader (Cellular Technology Ltd., Cleveland,OH). A response was considered positive only if there were greater than 50 spots per million cells, and if responses were greater than twice those recorded in the media controls. Technicians were blinded as to the vaccination group of the animals whose cells they were testing.

### Malaria test antigens for ELIspot

In all three studies we tested ELIspot response to the PkCSP antigen using pools of synthetic 20 aa peptides overlapping by 10 aa and spanning the antigen. The concentration of each peptide in the pool was 10 µM. In experiment #1, response to PkMSP1 was tested using an *E. coli* derived PkMSP1 19kD fragment tested at 2 µM (a gift of Dr. Sanjai Kumar). This 19kD fragment is contained within the 42kD PkMSP1 fragment used in our Pk vaccines. In experiment #3, we tested response to the PkAMA-1 antigen using pools of synthetic 15 aa peptides overlapping by 11 aa and spanning the antigen. The concentration of each peptide in the pool was 2 µM.

Statistical Methods: For each of the three separate experiments, we used Student's T test and Fisher's Exact Test for analysis of continuous and dichotomous variables respectively. For analysis of combined data from the three experiments in the final figure, we used a simple linear regression model.

## Results

Experiment # 1. Our goal in experiment #1 was to compare three priming regimens: 3 injections of DNA in PBS, 1 injection of DNA in PBS, and 3 injections of DNA formulated on PLG microspheres. All three groups received a boost with the same COPAK virus vaccine 16 weeks after the last DNA immunization. A control group received a mock vaccine, with control plasmid on PLG microspheres and a boost with control COPAK virus (Table 1). The three experimental groups were immunized with DNA encoding four Pk antigens, PkCSP, PkSSP2, PkAMA-1, and PkMSP1. Each of these four DNA plasmids was given into a separate im site (the left and right triceps and the left and right quadriceps) to preclude any interactions at the injection sites. Four weeks after the COPAK dose, all animals were challenged with 100 Pk sporozoites given iv and followed for the development of blood stage parasitemias. Blood was taken for in vitro studies before the first injection, four weeks after each DNA vaccination, and prior to challenge.

**Table 1:** Immunization schedule for Experiment #1

Group	Vaccine Name	Dose 1	Dose 2	Dose 3	Dose 4	Challenge
		wk 0	4	8	24	28
1	DNA Pk4×3/COPAK	DNA	DNA	DNA	COPAK	X
2	DNA Pk4×1/COPAK			DNA	COPAK	X
3	PLG Pk4×3/COPAK	DNA on PLG	DNA on PLG	DNA on PLG	COPAK	X
4	Control	Control DNA on PLG	Control DNA on PLG	Control DNA on PLG	Control COPAK	X

Five monkeys in each of four vaccine groups were immunized and challenged with Pk malaria sporozoites. ‘Pk4’ refers to four Pk antigens (PkCSP, PkSSP2, PkAMA1, and PkMSP1). ‘DNA’ indicates plasmid vaccines in PBS. ‘PLG’ indicates plasmid vaccines complexed to PLG particles. ‘COPAK’ indicates vaccinia encoding malaria antigen. Animals in the Control group received a mock vaccine consisting of plasmid without malaria inserts complexed to PLG particles followed by parental COPAK without malaria inserts. (See text for more details).

**Figure 1:**
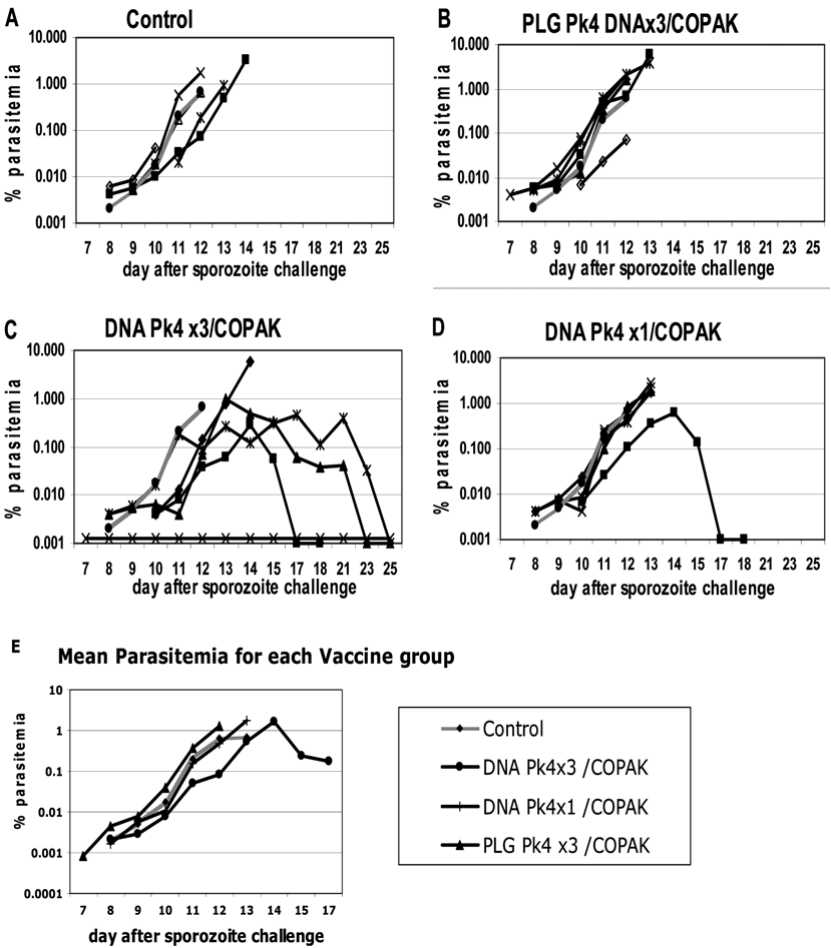
Panels A–D show the % parasitemia for individual monkeys by vaccine group according to the day after sporozoite challenge for Experiment #1. Data shows the first day parasites were detected and continues until the animal was drug treated when parasitemia exceeded 1% ( 3 animals inadvertently treated at lower parasitemias have open symbols). For comparison, in each panel the grey line shows the mean parasitemia for the Control group. In panel C, one animal never became parasitemic as indicated by x-x. Panel E shows the geometric mean parasitemias for vaccine groups for all days in which at least three animals had not been drug treated. (The monkey from the DNA Pk4×3/COPAK group which did not become infected was excluded from the average).

Figure 1 shows the parasitemias for individual monkeys plotted by vaccine group. We followed each animal until its parasitemia exceeded 1% at which time it was treated with anti-malarial drugs. Three animals, two in the control group and one in the PLG DNA group were inadvertently treated for malaria at parasitemias lower than 1% (these are shown with open markers). Figure 1 panel E shows the geometric mean parasitemia for each vaccine group for each day that at least three animals remained untreated for malaria. Table 2 summarizes the information on the first day parasites appeared in the blood and the day they reached 1%.

**Table 2:** Summary of Parasitemia Data in Experiment #1

Vaccine	Day 1^st^ parasitemia	Mean day 1^st^ parasitemia	Day >1% parasitemia	Mean Day >1% parasitemia
DNA Pk4×1/COPAK	8		13	
	8		13	
	9	8.8	13	13+
	9		13	
	10		never	
DNA Pk4×3/COPAK	8		never	
	8		never	
	10	9.0+	never	14+ *
	10		14	
	never		-	
PLG Pk4×3/COPAK	7		12	
	8		12	
	8	8.2	12	12.25
	8		13	
	10		unknown	
Control	8		unknown	
	8		14	
	9	9.0	unknown	13
	9		12	
	11		13	

‘Day 1^st^ parasitemia’ is the day after sporozoite challenge when parasites were first seen on malaria smear. ‘Day >1% parasitemia’ is the day of drug treatment (‘unknown’ means that monkeys were drug treated before reaching 1% parasitemia). Priming with 3 doses of DNA in PBS gave better protection than priming with 1 dose of DNA in PBS or 3 doses of DNA on PLG (*p = 0.04 Fisher's Exact test).

The DNA priming method strongly affected the efficacy of the DNA/COPAK vaccine. The group of monkeys primed with three doses of DNA in PBS were best protected (p<0.05 Fisher Exact test, Table 2). Of the 5 animals primed with three doses of DNA in PBS one monkey never developed blood stage parasites presumably due to complete inhibition of malaria in the sporozoite and liver stages. The other 4 animals developed parasitemias at the same time as the Control animals but as a group had lower geometric mean parasitemia on all days, although this did not reach statistical significance (Figure 1 panel E). Growth of parasites was similar in all groups up until days 12–13 when growth slowed in 3 of 4 infected animals in the DNA Pk4×3/COPAK group. These 3 animals controlled their blood stage infections below 1% parasitemia without drug therapy. In contrast, all 5 monkeys receiving only a single dose of DNA in PBS developed parasitemia, and 4 of 5 reached parasite levels over 1% and were treated. All 5 monkeys receiving 3 doses of DNA on PLG microspheres all became infected, and the 4 animals followed to the end of the study developed parasitemias over 1%.

**Figure 2:**
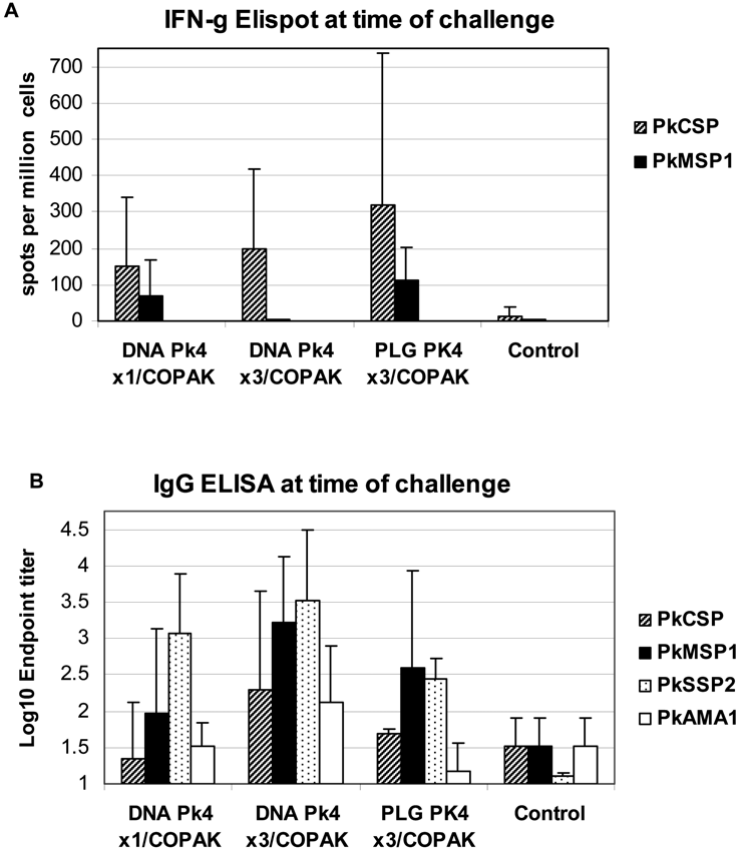
Panel A. Interferon-γ ELIspot was tested for only two antigens: PkCSP and PkMSP1. Spots in medium controls were subtracted from antigen test wells, and the results averaged. Priming with DNA on PLG gave a stronger interferon-γ response than DNA in PBS but this was not significant (p = 0.58). Panel B. Geometric mean antibody titers by ELISA at time of sporozoite challenge for each of the four malaria vaccine antigens. Priming with 3 doses of DNA gave higher antibody levels than did a single DNA priming dose for PkCSP, PkMSP1 and PkAMA1 antigens (p<0.05). Priming with 3 doses of DNA in PBS produced higher serum antibody titers than did priming with 3 doses of DNA on PLG for PkCSP, PkSSP2, and PkAMA1 antigens (p<0.05).

Figure 2. summarizes the immune responses for individual monkeys in experiment #1. Both ELISA's and INF-g ELIspot assays were run on frozen samples taken before immunization, four weeks after the final DNA immunization, on the day of COPAK boost, and four weeks after boost at the time of malaria challenge. No significant antibody or IFN-g responses were seen at baseline or after the priming immunizations (data not shown). After COPAK boost, substantial T cell and antibody responses appeared.

Figure 2 panel A plots the interferon-γ ELIspot responses after COPAK boost for the two antigens tested, PkCSP and PkMSP1. The group primed with DNA on PLG microspheres had the highest average ELIspot responses for both antigens however this was not statistically significant (p = 0.58 Student's T test) because of the large variation in responses between animals.

Figure 2 panel B shows antibody responses to each of the four vaccine components after the COPAK boost. The group of animals primed with three doses of DNA in PBS had the highest geometric mean titers of serum IgG for all four antigens. For most antigens, these differences were statistically significant (see legend).

Experiment #2 was designed to answer two questions. Firstly, can the PkCSP vaccine alone or with the PkSSP2 vaccine protect as well as the four antigens Pk4 vaccine (with PkCSP, PkSSP2, PkAMA-1, and PkMSP1)? Secondly, does increasing PkCSP DNA doses during priming from 0.5 mg to 5.0 mg lead to stronger immune responses or enhanced protection after COPAK boost? Table 3 gives the immunization schedule for experiment #2. As in experiment #1, each component of the DNA vaccine was given im by a single 1 ml injection into a separate limb. The one exception was the group receiving 5 mg of PkCSP DNA, which received two injections of 1 ml into each thigh because of concerns about concentration and viscosity of the DNA vaccine. In experiment #2 monkeys were followed until parasitemias exceeded 2% and then treated with anti-malarial drugs.

**Table 3:** Immunization schedule for Experiment #2

Group	Vaccine Name	DNA amount	Dose 1	Dose 2	Dose 3	Dose 4	Challenge
			wk 0	4	8	24	28
1	DNA Pk4×3/COPAK	0.5 mg of 4 DNAs	DNA	DNA	DNA	Pk4 COPAK	X
2	PkCSP 0.5 mg/COPAK	0.5 mg	DNA	DNA	DNA	PkCSP COPAK	X
3	PkCSP 5.0 mg/COPAK	5.0 mg	DNA	DNA	DNA	PkCSP COPAK	X
4	PkCSP+PkSSP2/COPAK	0.5 mg of 2 DNAs	DNA	DNA	DNA	PkCSP+PkSSP2 COPAK	X
5	Control	2.0 mg	DNA	DNA	DNA	Control COPAK	X

Five monkeys in each of five vaccine groups were immunized and challenged with Pk malaria sporozoites. The four antigen vaccine given Group 1 was identical to that given to Group 1 in Experiment 1. Group 2 received only the PkCSP components of the vaccine given to Group 1. Group 3 received a ten-fold larger dose of the PkCSP DNA than Group 2 and the same dose of PkCSP COPAK. Group 4 received only the PkCSP and PkSSP2 components of the vaccine given to Group 1. Group 5, the Control group, received a mock vaccine consisting of plasmid DNA without malaria antigen inserts followed by parental COPAK without malaria inserts. (See text for more details).

Experiment #2. Vaccine effects on parasitemia.

**Figure 3:**
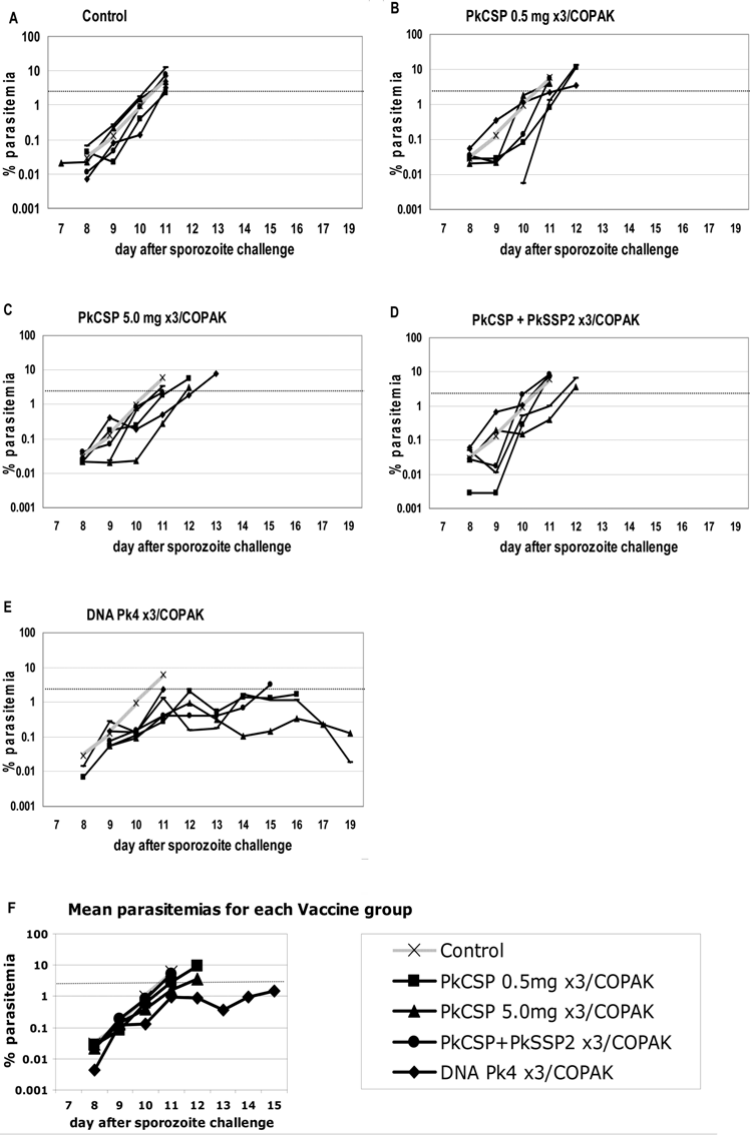
Panels A–E show the % parasitemia for individual monkeys by vaccine group according to the day after sporozoite challenge for Experiment #2. Data shows the first day parasites were detected and continues until the animal was treated with anti-malarial drugs. For comparison, in each panel the grey line shows the mean parasitemia for the Control group. Panel F shows the geometric mean parasitemias for all the vaccine groups for all days in which at least three animals had not been drug treated.

Figure 3 panels A–E show the parasitemias for individual monkeys in this experiment while panel F shows the geometric mean parasitemias for each group. Table 4 summarizes information on the day parasites were first detected following challenge, and the day when each animal's parasitemia exceeded 2%. Figure 3 panel A shows parasitemias in Control monkeys immunized with the mock vaccine. All Control animals had parasites detected in the blood on day 7 or 8 after sporozoite challenge, showed logarithmic parasite growth, and all were treated on day 11 after they exceed 2% parasitemia. Panel E shows parasitemias for the monkeys receiving the Pk4 DNA×3/COPAK vaccine (given identically as in Experiment #1). In Experiment #2, no monkeys were sterilely protected however the vaccine had a substantial impact on infection. There was a statistically significant one day delay in the appearance of the first parasites compared to Control (p = 0.03, Fisher Exact test). Geometric mean parasitemias were lower in the Pk4 DNA×3/COPAK vaccine group with statistically significant differences from Control monkeys on days 8, 10, and 11 (p<0.05, Student's T test). Parasite numbers increased at similar rates in all groups until day 11, when 4 of 5 of the monkeys receiving the Pk4 DNA/COPAK vaccine showed a slowing of parasite growth. As a result, 2 of 5 monkeys in this vaccine group never reached 2% parasitemia, and 2 animals had a delay of several days until treatment was required, a statistically significant difference from Control animals (p<0.001, Fisher Exact test).

**Table 4:** Summary of parasitemia data in Experiment #2

Vaccine	Day 1^st^ parasite	Mean day 1^st^ parasite	Day >2% parasitemia	Mean Day >2% parasitemia
PkCSP 0.5 mg×3/COPAK	8		11	
	8		11	
	8	8.4 *	12	11.6
	8		12	
	10		12	
PkCSP 5.0 mg×3/COPAK	7		11	
	8		11	
	8	8.0	12	11.8
	8		12	
	9		13	
PkCSP+PkSSP2×3/COPAK	7		11	
	7		11	
	7	7.4	11	11.4
	8		12	
	8		12	
DNA Pk4×3/COPAK	8		11	
	8		15	
	8	8.4*	16	14+**
	9		never	
	9		never	
Control	7		11	
	7		11	
	7	7.4	11	11
	8		11	
	8		11	

‘Day 1^st^ parasite’ is the day after sporozite challenge when a parasite was first seen on malaria smear. ‘Day >2% parasitemia’ is the time of drug treatment. The group receiving the four antigen vaccine and the single antigen PkCSP vaccine had a delay in the appearance of parasites in the blood (*p = 0.03 vs Control group, Fisher's Exact Test). The group receiving high doses of PkCSP DNA priming was not as well protected. Co-immunization with PkSSP2 and PkCSP was worse than immunization with PkCSP alone (p = 0.03). Only the monkeys receiving all 4 antigens were protected against high parasitemias (**p<0.001 vs Control group).

Figure 3 panels B, C, and D and Table 4 show parasitemias for animals receiving the PkCSP vaccine alone, the high DNA dose PkCSP vaccine, and both the PkCSP and PkSSP2 vaccines. Monkeys receiving the PkCSP 0.5 mg DNA/COPAK vaccine showed a one day delay in the time to first parasitemia as compared to the control group (p = 0.03, Fisher Exact test). Animals receiving the ten-fold higher doses of the DNA vaccine did not show any increased time to first parasitemia compared to monkeys given lower doses. Curiously, monkeys receiving concurrent vaccinations with both the PkCSP and the PkSSP2 antigens were less protected than animals receiving the PkCSP vaccine alone (p = 0.03, Fisher Exact test), showing first parasites in the blood the same day as the Control group.

Of the 15 animals receiving only the pre-erythrocytic PkCSP and/or PkSSP2 vaccines, none was able to control its blood stage parasite growth and all needed treatment for parasitemias over 2% by day 13. This contrasts with the monkeys receiving the 4 antigen vaccine which included PkAMA1 and PkMSP1 (p<0.001 Fisher's Exact Test). Of these 5 monkeys, 2 animals controlled their parasitemia without drug treatment, 2 had long delay until parasitemia reached treatment levels, while one was treated on day 11.

**Figure 4:**
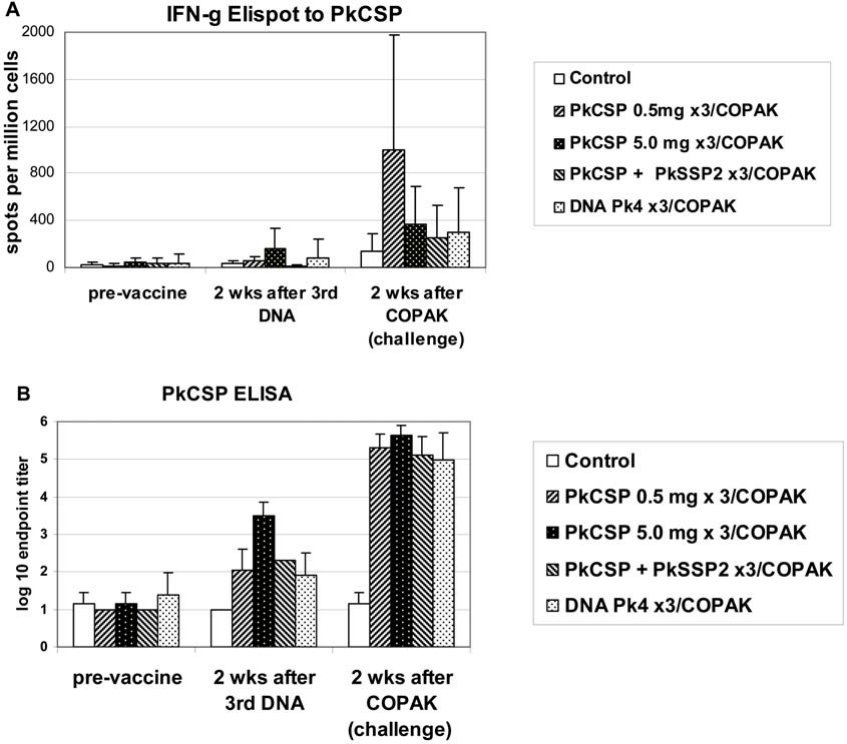
Panel A shows interferon-γ ELIspot response to PkCSP by vaccine group prior to vaccination, after the third DNA vaccination, and after the COPAK boost. After the third DNA vaccination, the PkCSP 5.0 mg group has the highest response but this is not significantly greater than the PkCSP 0.5 mg group (p = 0.11). After the third DNA vaccination monkeys receiving both PkCSP and PkSSP2 immunizations had significantly lower responses to PkCSP than did animals receiving only PkCSP DNA (p = 0.04). After the COPAK boost, monkeys primed with low dose PkCSP 0.5 mg DNA had the highest response interferon-γ response (p = 0.08 vs high dose PkCSP, p = 0.04 vs Pk4 vaccine, p = 0.04 vs PkCSP+PkSSP2). Panel B shows IgG responses at the three time points. After three DNA vaccinations, serum titers were highest in the high dose DNA PkCSP 5.0 mg group (p = 0.01). After boosting with recombinant COPAK virus, monkeys primed with the high dose DNA had the highest serum IgG titers but this was not significantly different from the other vaccine groups.

Figure 4. shows immune responses of animals to the PkCSP antigen in Experiment #2. Interferon-γ ELIspot T cell responses to PkCSP were measured in all animals at baseline, four weeks after the third DNA injection, and two weeks after the COPAK boost. After three DNA immunizations only weak T cell responses were detected (Figure 4 panel A). Animals receiving three 5.0 mg doses of PkCSP plasmid had slightly higher numbers of INF-γ producing T cells than did animals receiving 0.5 mg doses (p = 0.11). Monkeys receiving 0.5 mg doses of both the PkCSP and PkSSP2 DNA vaccines given in opposite legs (mean 16 spots/million sd = 11) had lower levels of PkCSP specific interferon-γ responses than monkeys receiving 0.5 mg PkCSP DNA alone (mean 54 spots/million sd = 36) (p = 0.04, Student's T test).

After boosting with COPAK vaccines, T cell responses to PkCSP increased in all groups. Monkeys primed with 0.5 mg PkCSP DNA had the highest T cell response but this was not significantly different than animals receiving the higher 5.0 mg dose ( p = 0.08). Animals receiving the 2 antigen PkCSP and PkSSP2 vaccine (mean 257 spots/million sd = 271) or the 4 antigen Pk4 DNA vaccine (mean 301 spots/million sd = 167) had lower interferon-γ responses than did animals receiving the single antigen 0.5 mg PkCSP vaccine (mean 997 spots/million sd = 981) (p = 0.04, Fishers Exact test).

Figure 4 panel B shows antibodies to PkCSP in experiment #2. Four weeks after the third DNA immunization, the anti-PkCSP titers were highest in the monkeys receiving the 5.0 mg plasmid dose (p<0.05) although titers were still modest. After the COPAK boost, all vaccine groups had high antibody titers to PkCSP. Although monkeys primed with the 5 mg DNA had the highest titers after boost, this was not statistically different from monkeys primed with 0.5 mg DNA (p = 0.28). Antibodies to PkCSP were the same in groups receiving 0.5 mg of PkCSP DNA alone, or simultaneous vaccination with 0.5 mg of PkSSP2 DNA.

Very large PkCSP DNA doses gave higher immune T cell and antibody responses after DNA vaccination. However, these improved responses after DNA priming did not translate into improved immune response after the PkCSP COPAK boosting. Neither did the animals receiving high dose DNA priming show any improved protection against malaria as compared with those receiving low dose priming.

Simultaneous vaccination with PkSSP2 DNA into a separate limb seems to have inhibited interferon-γ T cell responses to PkCSP DNA. This is seen in a lower PkCSP specific interferon-γ response after the third DNA dose, as well as after the COPAK boost. These lower responses were mirrored in a complete lack of protection in the PkCSP+PkSSP2 group. We had hoped that using two pre-erythrocytic antigens would improve protection, but it appears that a negative interaction is occurring when animals are exposed to these two antigens. Interestingly, this interaction affected interferon-γ responses but did not affect antibody responses to PkCSP.

Finally, only animals receiving the four antigen vaccine including the blood stage antigens PkMSP1 and PkAMA-1 were able to control their malaria infections once organisms appeared in the blood. This is strong evidence that this blood stage protection is due to the antigen specific responses to these two proteins induced by genetic vaccination. In the absence of a group receiving only the PkMSP1 and PkAMA1 vaccines, we do know if the two pre-erythrocytic stage antigens contribute to this blood stage efficacy, or if protection against blood stage infections would be seen with a vaccine containing only PkMSP1 and PkAMA1.

Experiment #3. In this experiment, we compared 7 week and 21 week intervals between the third DNA dose and COPAK boost using the Pk4×3DNA/COPAK vaccine. (A 16 week interval was used in Experiments #1 and #2. It was our intention to compare 7 and 16 week intervals in this experiment, however logistical problems caused a delay). The vaccination and challenge were as in Experiments #1 and #2. There were 4 animals in the 7 week interval group, and 5 animals in the other two groups.

**Figure 5:**
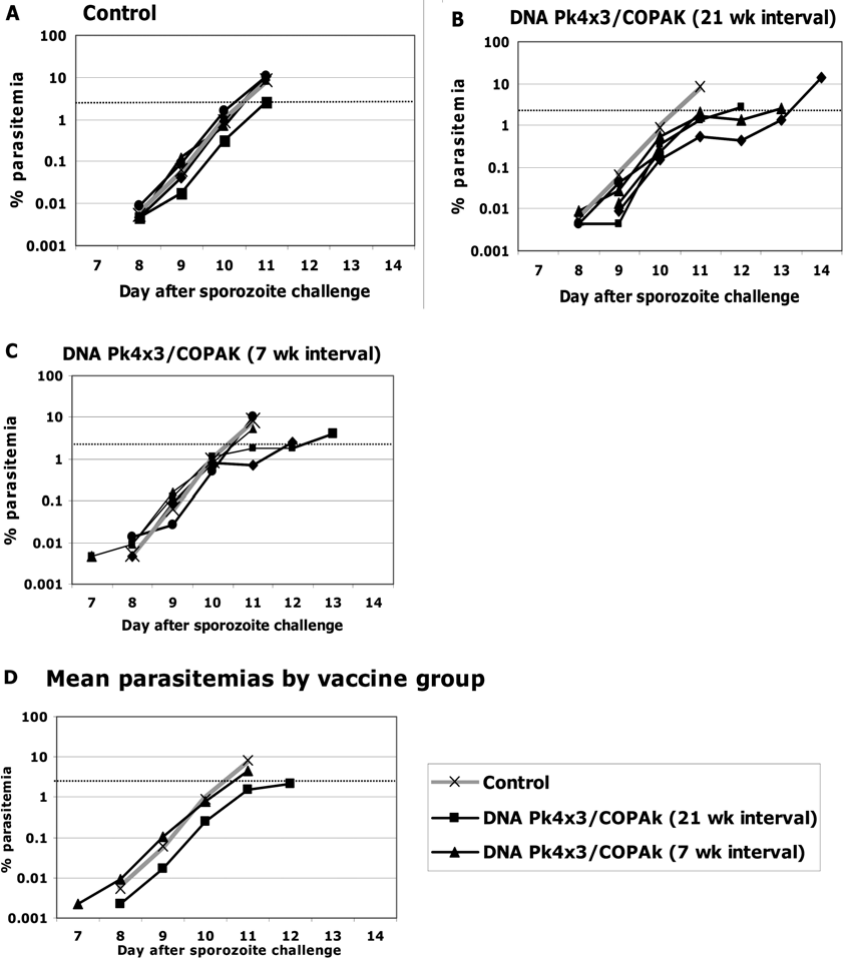
Panels A–C show the % parasitemias for individual monkeys by vaccine group according to the day after sporozoite challenge for Experiment #3. Data shows the first day parasites were detected and continues until the animal was treated with anti-malaria drugs. For comparison, in each panel the grey line shows the mean parasitemia for the Control group. Panel D shows the geometric mean parasitemias for all the vaccine groups for all days in which at least three animals had not been drug treated. For each day 8–11 the mean parasitemia for the group receiving the booster dose at the 21 week interval was lower than the other groups (p<0.05, Student's T test).

Figure 5 panels A–C show the parasitemias for individual monkeys in Experiment #3 and panel D shows the geometric mean parasitemias for each group. All Control and vaccinated monkeys developed blood stage infections on days 7–9 after sporozoite injection. Neither of the vaccine groups had a delay to day of first parasitemia compared to the Control group. However, compared to the Control and 7 week interval groups, the Pk4 DNA×3/COPAK 21 week group had a lower mean parasitemia on days 8–11 (p<0.05 by Student's T test) and a one day delay to day >2% parasitemia compared to the Control group (p = 0.023 Fishers Exact test). All monkeys required drug treatment and there were no self-cures. Immune responses at the time of challenge showed no statistically significant differences between the groups with 7 or 21 week intervals in antibody titers by ELISA or in interferon-γ ELIspots, although there were slightly higher T cell responses in the group with the longer interval (data not shown).

Conclusions from Experiment 3. Comparing the groups with 7 and 21 week intervals, the longer interval resulted in lower mean daily parasitemias and a delay in reaching 2% parasitemia. Although the group with 21 week intervals was not as well protected as were the groups with 16 week intervals in Experiments #1 and #2, we are loath to conclude that one is interval is superior as the animals in the three experiments were challenged at different times with different batches of sporozoites.

Correlation of Immune Responses to PkCSP with parasitemia in experiments 1, 2, and 3.

The small numbers of animals in each of the three experiments lead us to combine the results for correlation of immune response at the time of sporozoite challenge and protection against malaria. There was no correlation between antibody titers to any vaccine antigen with day of first parasitemia or day when the treatment threshold of parasitemia was reached (data not shown). Interferon-γ ELIspot responses for PkMSP1 and PkAMA1 were only tested in single experiments and the small samples size provides insufficient statistical power to attempt correlations with protection. However, interferon-γ ELIspot responses to PkCSP were measured for all animals in these three experiments and they showed a negative correlation with the appearance of first parasites in the blood.

**Figure 6:**
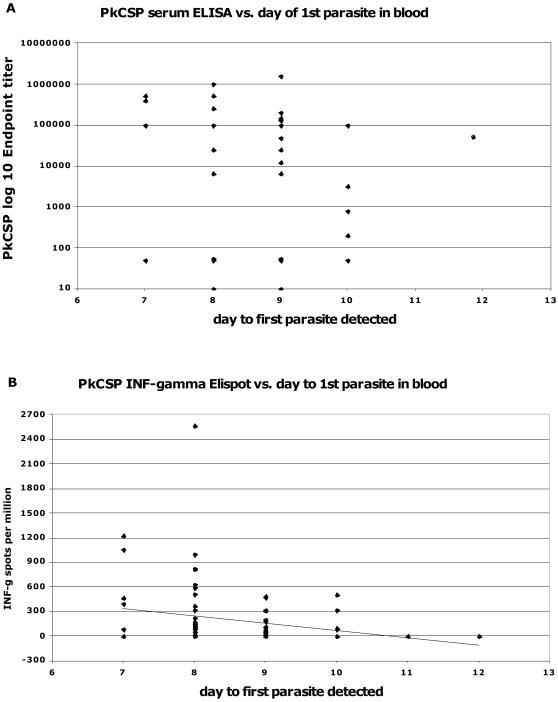
Immune response to PkCSP antigen at time of challenge for individual animals by day to first parasite seen in blood. Data from Experiments #1, 2, and 3 are plotted. Data from Control animals are not included. Panel A shows PkCSP endpoint ELISA titers where there is no significant correlation. Panel B shows PkCSP interferon-γ ELIspot titers with a linear regression line included. There is a significant negative correlation (p = 0.04), animals with lower ELIspot responses having longer times to the appearance of first parasite in the blood.

Figure 6 shows this surprising result. We have included all animals from Experiments 1, 2, and 3 that were immunized with any vaccine containing PkCSP (i.e. Control animals are excluded). In panel A, we have plotted the PkCSP antibody titer on day of challenge vs. day of first parasitemia. There is no correlation between antibody titer and time to parasitemia. Figure 6. panel B shows a plot of interferon-γ ELIspot to the PkCSP antigen for the same animals. Here there is a statistically significant negative association, with animals having stronger responses showing parasites earlier in the blood (p = 0.04, linear regression model). This negative correlation of PkCSP interferon-γ response and time to first parasite detected is also true for each of the three experiments analyzed individually, although the smaller numbers do not lead to statistically significant results (data not shown).

## Discussion

Our goal in developing a primate malaria vaccine model is to define the important parameters governing vaccine efficacy prior to designing malaria vaccines studies in humans. We draw four lessons from the malaria vaccine experiments presented in this paper: 1) The timing, number and formulation of the DNA injections is critical to the protective efficacy of the DNA/COPAK vaccine, 2) DNA/COPAK malaria vaccines can provide protection against both pre-erythrocytic and blood stage malaria infection, 3) the antigens can interfere with each other in a multi-antigen vaccine even when they are given at separate injection sites, and 4) we have not yet identified the immune responses which protect these immunized monkeys against malaria.

1) In this prime/boost vaccine model in rhesus monkeys, the dose, number, and formulation of DNA injections are critical for vaccine efficacy. However, the absolute quantity of DNA injected in priming before COPAK boost was less important. In experiment #1, the Pk4 DNA×3/COPAK vaccine with 3 monthly DNA doses provided a moderate level of protection against sporozoite challenge, similar to what we have previously reported [45], [46]. Priming with only a single dose of DNA plasmid led to similar interferon-γ responses, but reduced antibody levels and less vaccine efficacy. Formulating the three DNA doses on PLG microspheres enhanced the interferon-γ responses to the vaccine but also reduced antibody responses and efficacy. In Experiment #2, large doses of PkCSP plasmid gave higher immune responses before the viral boost, but this did not translate into better immune responses or protection after boost. In Experiment #3, shorter intervals between prime and boost led to less protection. The lesson for human vaccine development would seem to be that much effort should be spent in optimizing priming regimens in human DNA prime/viral boost trials, with emphasis on finding optimum intervals [52] and formulations rather than escalating DNA quantities.

2) In these experiments, only animals receiving the four antigen vaccines were able to control parasite growth after parasites were detected in the blood. Of the four malaria antigens in this vaccine, PkCSP and PkSSP2 are primarily expressed on sporozoites [53]–[57] and may be present in early stage infected liver cells. PkAMA1 and PkMSP1 are expressed in infected red cells and late stage infected liver cells [58] , although there is some data indicating that AMA1 is also expressed in sporozoites [54], [59]. Animals receiving vaccines with the PkCSP and or PkSSP2 antigens demonstrated a delay in appearance of parasites in the blood, consistent with killing of sporozoites or infected liver cells and development of fewer liver schizonts [60]. However, in animals which only received vaccines with PkCSP or PkSSP2, once red cells became infected there were logarithmic increases of parasites. Only animals receiving vaccines containing the PkAMA1 and PkMSP1 antigens could control their red cell infections below levels requiring drug treatment. This is strong evidence that the immune response to these blood stage antigens induced by a DNA/viral vaccine was effective in limiting parasite growth.

Most work on blood stage vaccines, including AMA1 and MSP1, has focused on protein vaccines where the correct folding and glycosylation of antigens has been critical for the development of inhibitory antibodies and protection [11]–[23]. Both the DNA plasmid and viral components of our vaccine are based on parasite DNA sequences which the mammalian cell uses to produce antigenic proteins using its intrinsic controls for folding, post-translational modifications, and degradation. These vaccine antigens may or may not have the same folding and glycosylation as they have in the parasite. We believe that the lesson for human vaccine development is that blood stage antigens can be protective in genetic vaccines, and should be included despite the limited ability to control their final three dimensional structures. It is not clear that the protective effect of these genetic blood stage vaccines is due to the direct induction of inhibitory antibodies. The typical course of parasitemia in our vaccinated animals is a logarithmic rise in blood stage parasites for several days which then slows, plateaus, and decreases. This is not the pattern expected from a vaccine which induces high levels of protective antibody, where one might expect a uniform slow rate of parasite growth. An alternative hypothesis is that the genetic vaccines have induced helper T cell responses to the blood stage antigens, which allows the host to rapidly produce inhibitory antibodies after antigens appear in the blood. We are currently examining this hypothesis by comparing the inhibitory effects of serum antibody in immunized animals both before and after challenge.

3) Experiment #2 provided evidence of antigen interference between PkCSP and PkSSP2. Our unexpected finding is that administration of the PkSSP2 DNA vaccine in the left leg diminished the interferon-γ T cell responses and efficacy of the PkCSP DNA vaccine given in the right leg. However, antibody responses to PkCSP DNA vaccination were not affected. Monkeys in the four antigen vaccine group, which received the PkCSP and PkSSP2 DNA vaccines in the legs but also received the PkAMA1 and PkMSP1 DNA vaccines in the arms, also had lower interferon-γ responses to PkCSP than the group receiving PkCSP alone. We do not pretend to understand these phenomena. Previously, inhibition between DNA vaccines has been described when the vaccines were given together in the same syringe, and was thought to be due to competition between plasmids within mammalian cells [61]–[63]. To avoid such competition, we administered each of our DNA vaccine antigens into muscles on different limbs. However, this is not the first instance of undesirable interactions between CSP and SSP2 vaccines [64]. If the PkSSP2 antigen is detrimental, it might be interesting to test a trivalent vaccine with PkCSP, PkMSP1, and PkAMA1. Similarly, it will be important to test for inhibition in multi-antigen human malaria vaccines.

4) The most surprising result of our experiments is the negative correlation between interferon-γ responses to the PkCSP and time to first parasite detected in the blood. Why should stronger T cell responses lead to parasites appearing sooner? We believe that T cell responses are protective but that the protective T cell responses are in some way reciprocal to the T cell responses we are measuring. Our assay measures interferon- γ using circulating lymphocytes in an overnight stimulation assay. This assay primarily measures responses from CD4^+^ T cells which are activated effector cells [51]. In humans immunized with pre-erythrocytic vaccine antigens, overnight ELIspot assays which measure circulating active effector cells correlate poorly with protection, but multi-day cultures which measure effector memory cells have a stronger association with protection from malaria challenge [65]. It is likely that the true cells which are killing parasites are immune T cells residing in the liver, which are difficult to sample [66]. Perhaps the puzzling inverse correlation we have described is because the number of antigen specific effector cells remaining in the circulation is inversely related to the numbers of protective immune cells which have homed to the liver. We are developing methods for directly measuring immune responses in monkey liver so that we can test this hypothesis in future experiments. The implication of this finding for human vaccine studies is that we have not found an immune correlate of protection. Until we do, pre-erythrocytic vaccines should not be optimized to produce specific immune responses but instead protection against sporozoite challenge must continue to be the standard.
